# Morpho-Physiological Traits and Oil Quality in Drought-Tolerant *Raphanus sativus* L. Used for Biofuel Production

**DOI:** 10.3390/plants13121583

**Published:** 2024-06-07

**Authors:** Luciana Minervina de Freitas Moura, Alan Carlos da Costa, Caroline Müller, Robson de Oliveira Silva-Filho, Gabriel Martins Almeida, Adinan Alves da Silva, Elivane Salete Capellesso, Fernando Nobre Cunha, Marconi Batista Teixeira

**Affiliations:** 1 Laboratório de Ecofisiologia e Produtividade Vegetal, Instituto Federal de Educação, Ciência e Tecnologia Goiano, Campus Rio Verde, Rio Verde 75901-970, GO, Brazil; lucianaminervina@gmail.com (L.M.d.F.M.); carolinemulleram@gmail.com (C.M.); robsonagro2015@gmail.com (R.d.O.S.-F.); gabrielrvmartins@gmail.com (G.M.A.); adinan.alves64@gmail.com (A.A.d.S.); 2 Centro de Excelência em Agricultura Exponencial (CEAGRE), Rua das Turmalinas, 44—Vila Maria, Rio Verde 75905-360, GO, Brazil; marconi.teixeira@ifgoiano.edu.br; 3 Centro de Excelência em Bioinsumos (CEBIO), Rua 88, 30—Setor Sul, Goiânia 74085-010, GO, Brazil; 4 Laboratório de Ecologia Vegetal, Universidade Federal do Paraná—Centro Politécnico, 100, Curitiba 81530-000, PR, Brazil; elivanesc@gmail.com; 5 Laboratório de Hidráulica e Irrigação, Instituto Federal de Educação, Ciência e Tecnologia Goiano, Campus Rio Verde, Rio Verde 75901-970, GO, Brazil; fernandonobrecunha@hotmail.com

**Keywords:** water deficit, forage turnip, adaptation strategies, biodiesel

## Abstract

*Raphanus sativus* L. is a potential source of raw material for biodiesel fuel due to the high oil content in its grains. In Brazil, this species is cultivated in the low rainfall off-season, which limits the productivity of the crop. The present study investigated the effects of water restriction on the physiological and biochemical responses, production components, and oil quality of *R. sativus* at different development stages. The treatments consisted of 100% water replacement (control), 66%, and 33% of field capacity during the phenological stages of vegetative growth, flowering, and grain filling. We evaluated characteristics of water relations, gas exchange, chlorophyll *a* fluorescence, chloroplast pigment, proline, and sugar content. The production components and chemical properties of the oil were also determined at the end of the harvest cycle. Drought tolerance of *R. sativus* was found to be mediated primarily during the vegetative growth stage by changes in photosynthetic metabolism, stability of photochemical efficiency, increased proline concentrations, and maintenance of tissue hydration. Grain filling was most sensitive to water limitation and showed a reduction in yield and oil content. However, the chemical composition of the oil was not altered by the water deficit. Our data suggest that *R. sativus* is a drought-tolerant species.

## 1. Introduction

Forage turnip (*Raphanus sativus* L. var. *oleiferus* Metzg.) is an oilseed crop of the Brassicaceae family. It is cultivated in southern and central Brazil as a winter crop and follows major crops such as soybeans and maize [1]. For some time, *R. sativus* has been used as green manure, soil cover, nutrient recycling [2,3], soil relief, and crop rotation [4,5]. Its high erucic acid content makes it toxic and, therefore, unsuitable for human consumption [6,7]. Recent studies have identified *R. sativus* as a potential source of raw materials for the chemical industry [8,9]. Its grains have a high oil content (40–54%) and exhibit physicochemical properties important for biodiesel production [1,6,7,10]. The predominance of fatty acids such as oleic acid gives the oil a higher oxidation stability than soybean and sunflower oil; fewer solid residues are produced in biodiesel [1,11]. The low viscosity of the oil improves engine performance, a particularly important property for biodiesel production [6]. In addition, vegetable oils cause fewer pollutant emissions [11].

As with other crops, grain yield depends on the genotype and adequate light, temperature, and water supply. Water deficits are particularly responsible for low crop yields [12]. This problem is exacerbated by increasing climatic instability, which leads to prolonged droughts in agricultural areas [13].

In response to limiting water conditions, plants respond in a variety of ways, including stomatal closure, inhibition of the transcriptional activity of genes related to CO_2_ fixation [14], reduction in photochemical activity [15], and cell damage [16]. These responses affect plant growth and productivity [17] and alter oil content and quality [18].

The negative effects of water restriction depend on the duration, intensity, severity, and timing, as well as the genotype of the plant [19,20]. In water restriction, plants trigger processes that prevent dehydration, including osmotic adjustment [17,21] and root system growth [22]. Electron dissipation [23] and increased activity of antioxidant enzymes [24,25] are also important in preventing and mitigating cell damage. These results suggest that the ability to detect water deficits and trigger physiological and biochemical responses is critical for plant survival.

The use of *R. sativus* in industrial oil production depends on the technical, economic– and socio-environmental competitiveness of the plant [7]. In addition, agronomic factors (e.g., plant productivity, seasonality, adaptability) also influence the commercialization of this species as an energy crop. 

Since the current knowledge on water deficit and oil quality in *R. sativus* is incomplete, we aimed to measure the physiological and biochemical responses and production components, as well as the oil quality of plants at different developmental stages grown under water deficit conditions.

## 2. Results

### 2.1. Water Relations

**Figure 1:**
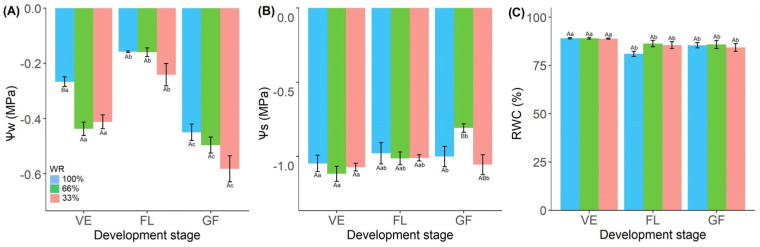
Predawn leaf water potential (Ψ_w_) (**A**), leaf osmotic potential (Ψ_s_) (**B**) and relative water content (RWC) (**C**) in *Raphanus sativus* plants exposed to three water levels: 100% (control), 66% and 33% of field capacity (FC) and at three phenological stages of development: vegetative (VE), flowering (FL) and grain filling (GF). The bars represent mean values ± SE (*n* = 4). Mean values with different letters differ significantly according to Tukey’s test (*p* ≤ 0.05). Uppercase letters show the differences between water replacement within each developmental stage, and lowercase letters show the differences between developmental stages in each water replacement.

Leaf water potential (Ψ_w_) decreases as soil water availability increases (Figure 1A), independently during the vegetative, flowering, and grain-filling stages (Figure 1A). We observed the lowest Ψ_w_ values in plants with 100% FC water saturation at the FL stage (Table S1). 

Osmotic potential (Ψ_s_) was similar between treatments, except at the GF stage, with lower values in 66% FC-treated plants (Figure 1B). Relative water content (RWC) was maintained above 80% under all treatment conditions but with lower values at the FL and GF stages (Figure 1C; Table S1).

### 2.2. Physiological Traits

**Figure 2:**
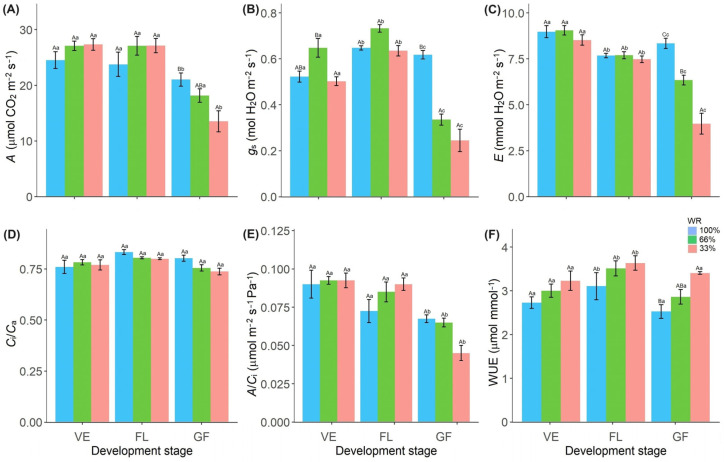
Photosynthetic net assimilation rate (*A*) (**A**), stomatal conductance (*g*_s_) (**B**), transpiration rate (*E*) (**C**), the ratio between internal and external CO_2_ concentration (*C*_i_/*C*_a_) (**D**), instantaneous carboxylation efficiency (*A*/*C*_i_) (**E**) and water use efficiency (WUE) (**F**) in *Raphanus sativus* plants exposed to three water levels: 100% (control), 66% and 33% of field capacity (FC) and at three phenological stages of development: vegetative (VE), flowering (FL) and grain filling (GF). The bars represent mean values ± SE (*n* = 4). Mean values with different letters differ significantly according to Tukey’s test (*p* ≤ 0.05). Uppercase letters show the differences between water replacement within each developmental stage, and lowercase letters show the differences between developmental stages in each water replacement.

Water deficit affected gas exchange in the phenological stages studied (Figure 2). At the GF stage, photosynthetic rate (*A*), stomatal conductance (*g*_s_), transpiration rate (*E*), and instantaneous carboxylation efficiency (*A*/*C*_i_) were lower in plants with a 33% FC water deficit (Figure 2A–C,E). The values of *g*_s_ and *E* were also lower in treated plants with 66% FC at the GF stage than in control plants (Table S1). 

The ratio between internal and external CO_2_ concentration (*C*_i_/*C*_a_) varied only depending on the phenological stage, with the highest values observed at the FL stage (Figure 2D). Water use efficiency (WUE) was higher in the FL stage, while values were lower in the control (Figure 2F; Table S1). 

**Figure 3:**
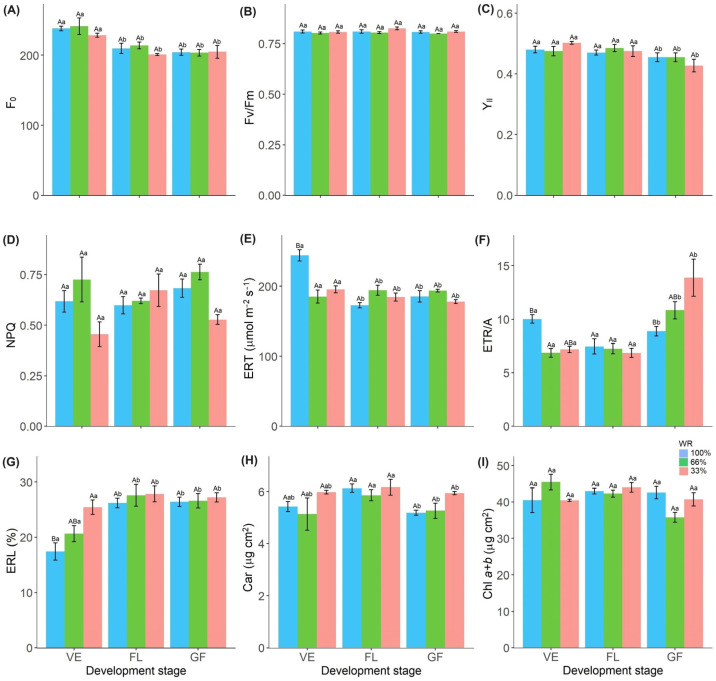
Minimum fluorescence (*F*_0_) (**A**), the potential quantum yield of PSII (*F*_v_/*F*_m_) (**B**), the effective quantum yield of PSII (Y_II_) (**C**), non-photochemical quenching coefficient (NPQ) (**D**), apparent electron transport rate (**E**) and the ratio between ETR and photosynthetic assimilation rate (ETR/A) (**F**), electrolyte leakage rate (ELR) (**G**), carotenoids (Car) (**H**), total chlorophyll content (Chl*a*+*b*) (**I**) in *Raphanus sativus* plants exposed to three water levels: 100% (control), 66% and 33% of field capacity (FC) and at three phenological stages of development: vegetative (VE), flowering (FL) and grain filling (GF). The bars represent mean values ± SE (*n* = 4). Mean values with different letters differ significantly according to Tukey’s test (*p* ≤ 0.05). Uppercase letters show the differences between water replacement within each developmental stage, and lowercase letters show the differences between developmental stages in each water replacement.

The minimum fluorescence (*F*_0_) varied according to the phenological stage, with the lowest values at the FL and GF stages (Figure 3A). The treatments did not affect the potential quantum yield of PSII (*F*_v_/*F*_m_) and averaged 0.81 throughout the experiment (Figure 3B). At the GF stage, the effective quantum yield of PSII (Y_II_) was reduced (Figure 3C; Table S1). 

The plants showed no differences in non-photochemical quenching (NPQ) (Figure 3D). The apparent electron transport rate (ETR) was lower in plants exposed to a 33% and 66% water deficit during the VE stage (Figure 3E). The water deficit led to a decrease in ETR/*A* during the VE stage. However, the same variable increased in the plants treated with 33% FC at the GF stage compared to the values of the control plants (Figure 3F).

The ELR was affected by the phenological stage, with the highest values observed at the FL and GF stages (Figure 3G). Water deficit resulted in higher ELR and was lower in the control plants (Figure 3G; Table S1). 

Carotenoid concentrations (Car) increased at the FL stage regardless of water condition (Figure 3H), while total chlorophyll concentrations (Chl*a*+*b*) did not differ by treatment (Figure 3I; Table S1).

### 2.3. Biochemical Traits

**Figure 4:**
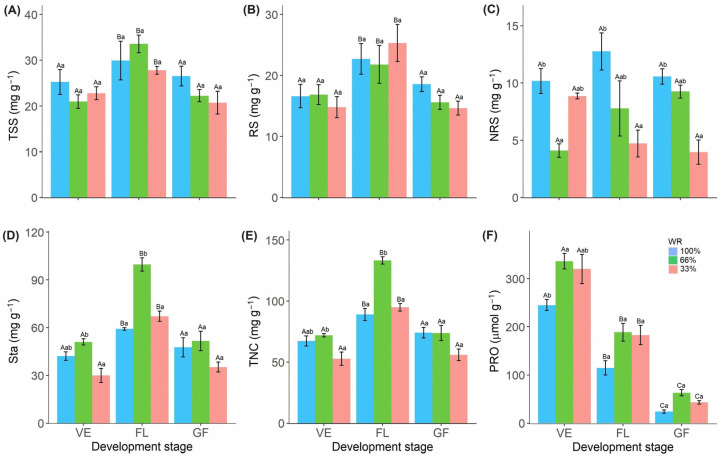
Total soluble sugars (TSS) (**A**), reducing sugars (RS) (**B**), non-reducing sugars (NRS) (**C**), starch (Sta) (**D**), total non-structural carbohydrates (TNC) (**E**), free proline (PRO) content (**F**) in *Raphanus sativus* plants exposed to three water levels: 100% (control), 66% and 33% of field capacity (FC) and at three different phenological stages of development: vegetative (VE), flowering (FL) and grain filling (GF). The bars represent mean values ± SE (*n* = 4). Mean values with different letters differ significantly according to Tukey’s test (*p* ≤ 0.05). Uppercase letters show the differences between water replacement within each developmental stage, and lowercase letters show the differences between developmental stages in each water replacement.

Total soluble sugars (TSS) and reducing sugars (RS) content were only affected by phenological stages and were higher at the FL stage (Figure 4A,B). The content of non-reducing sugars (NRS) was lower in the VE (66% FC), FL (33% and 66% FC) and GF (33% FC) stages compared to the respective controls (Figure 4C). Starch (Sta) and total nonstructural carbohydrate (TNC) contents were lower in the VE, FL, and GF stages in the plants with 33% FC water deficit than in the plants treated with 66% FC in FL and GF (Figure 4D,E; Table S1). The water deficit increased in Pro content, especially in the VE and FL stages (Figure 4F).

### 2.4. Component Production and Characterization of the Crude Oil

**Figure 5:**
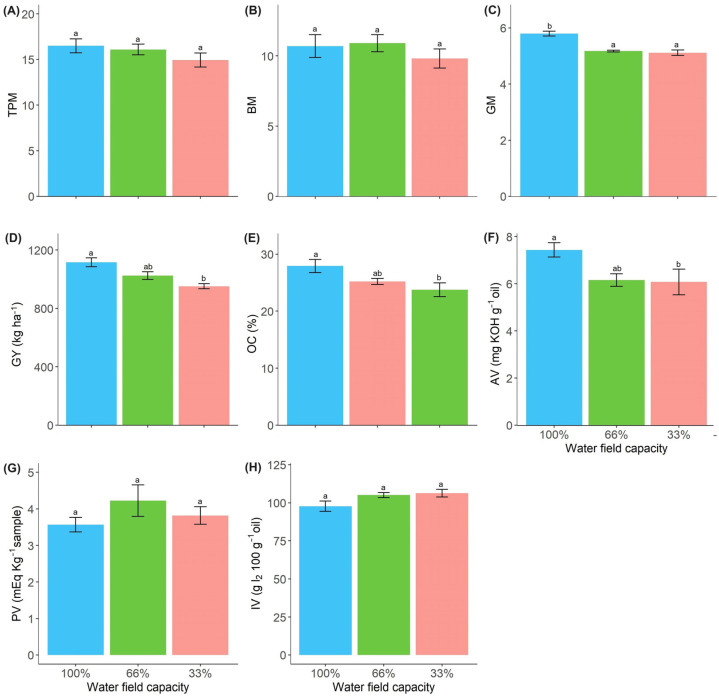
Total pod matter (TPM) (**A**), bark matter (BM) (**B**), grain matter of 100 pods (GM) (**C**), grain yield (GY) (**D**), grain oil content (OC) (**E**), acid value (AV) (**F**), peroxide value (PV) (**G**) and iodine value (IV) (**H**) in *Raphanus sativus* plants exposed to three water levels: 100% (control), 66% and 33% of field capacity (FC). The bars represent mean values ± SE [*n* = 4 (**A**–**D**), *n* = 8 (**E**), *n* = 12 (**F**–**H**)]. Mean values with different letters differ significantly according to Tukey’s test (*p* ≤ 0.05).

The treatments did not affect the total pod matter (TPM) and bark matter (BM) of 100 pods (Figure 5A,B). However, we observed higher grain matter (GM) and grain yield (GY) in plants treated with 100% FC (Figure 5C,D). 

The water deficit reduced grain oil content. Compared to the control, plants treated with 33% FC obtained the lowest oil content (OC) and AV values (Figure 5E,F). The water deficit did not affect the PV (Figure 5G) or IV (Figure 5H) crude oil content of *R. sativus* (Table S2).

**Figure 6:**
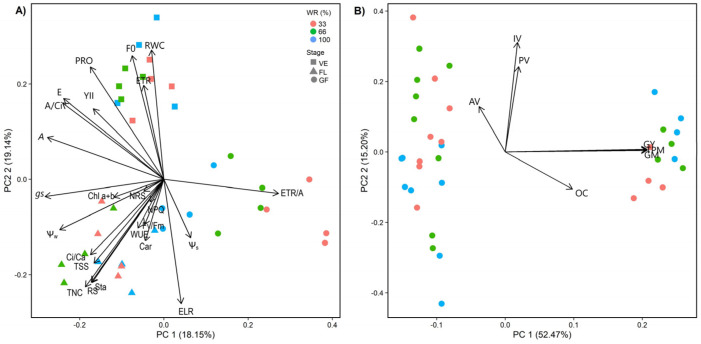
Biplot variation for physiological parameters (**A**) and productivity (**B**) in *Raphanus sativus* plants exposed to three water levels: 100% (control), 66%, and 33% of field capacity (FC). The bars represent mean values ± SE (*n* = 4 in (**A**); *n* = 12 in (**B**)). Pre-dawn leaf water potential (Ψ_w_), leaf osmotic potential (Ψ_s_), relative water content (RWC), photosynthetic net assimilation rate (*A*), stomatal conductance (*g*_s_), transpiration rate €, the ratio between internal and external CO_2_ concentration (*C*_i_/*C*_a_), instantaneous carboxylation efficiency (*A*/*C*_i_), water use efficiency (WUE), minimum fluorescence (*F*_0_), the potential quantum yield of PSII (*F*_v_/*F*_m_), the effective quantum yield of PSII (Y_II_), non-photochemical quenching (NPQ), apparent electron transport rate (ETR), the ratio between ETR and photosynthetic assimilation rate (ETR/*A*), electrolyte leakage rate (ELR), carotenoids (Car), total chlorophyll (Chl*a*+*b*), proline content (Pro), total soluble sugars (TSS), reducing sugars (RS), non-reducing sugars (NRS), starch (Sta), total non-structural carbohydrates (TNC), total pod matter (TPM), bark matter (BM), grain matter of 100 pods (GM), grain yield (GY), grain oil content (OC), acid value (AV), peroxide value (PV) and iodine value (IV).

We can observe segregation between water relations physiological and biochemical traits considering the stages of development (F_35,2_ = 10.58, *p* = 0.001). For water replacement, we found a difference between 33% and 100% (F_35,2_ = 4.90, *p* = 0.001). The first axis of the PCA explains 28.5% of the variation in the data, and the second axis corresponds to 19.1%. The first axis segregates the GF (●) from the FL (∆) and the VE (□), while the second axis segregates the VE and FL (Figure 6A). The VE was associated with the RWC, ETR, PRO, *F*_0_, and Y_II_. The FL was associated with Ψ_w_, *g*_s_, *F*_v_/*F*_m_, NRS, NPQ, WUE, Ci/Ca, Chl*a*+*b*, Car, TSS, RS, Sta and TNC (Figure 6A). The GF was associated with the ETR/A and Ψ_s_. We found no difference in productivity between water treatments (F = 0.00, *p* = 1.00; Figure 6B; Table S2).

## 3. Discussion

*Raphanus sativus* plants maintained their physiological processes under water restriction conditions (Table S1). This ability to physiologically adapt to drought may be crucial for survival, growth, and, consequently, crop yield in areas with limited water supply [17]. 

Under water stress conditions, reduced leaf water potential is usually accompanied by reduced stomatal conductance to minimize water loss through transpiration [26]. However, this was not observed in *R. sativus* plants, as stomatal conductance and transpiration rate were not altered during the initial phase of stress (Figure 2B,C); plants maintained a high relative water content in the leaves (Figure 1C). Water potential and relative water content are important physiological indicators of a plant’s water status [27]. Plants that are more resistant to water deficit have higher WUE than sensitive plants [20], as this allows higher photosynthetic rates with the least water loss [14], as observed in *R. sativus* grown under low water conditions (Figure 2A–F). The absence of photochemical damage (Figure 3B) is also related to the maintenance of hydration of *R. sativus* leaves. Zivcak et al. [28] observed a correlation between the relative water content reduction and the electron transport reduction. They increased non-photochemical extinction in wheat plants grown under limited water conditions. 

Maintenance of turgor under water stress partially occurs through osmotic adjustment by increasing Pro synthesis and protecting cell membranes from desiccation [17,21]. The Pro accumulation observed in *R. sativus* leaves (Figure 4F) suggests that it is a compatible solute, which has also been observed in chickpeas [29], fava beans [30], and soybeans [31]. It also suggests a role in antioxidant defense, as described in chickpeas [25], wheat [32], and rice [16]. In *R. sativus*, this was evidenced by the negative correlations between Pro and osmotic potential and ELR (Figure 6, Table S2). In addition, the increased energy requirement at the reproductive stage explains the decrease in Pro content at all developmental stages of *R. sativus* (Figure 4F).

Oxidative stress is usually described as a secondary effect of various abiotic stress factors that cause oxidation of photosynthetic pigments [24,33] and act on the antenna complex and reaction centers of photosystems. We observed the maintenance of the potential quantum yield of PSII, ETR (Figure 3B–E), photosynthetic pigments (Figure 3H,I), and ELR (Figure 3G) in conjunction with the photosynthetic rates (Figure 2), indicating the absence of oxidative damage and demonstrating the physiological drought-tolerance of *R. sativus*. Under high photochemical activity, carbohydrate production was maintained, contributing to the growth and productivity of *R. sativus* plants, even under limited water conditions.

During the GF stage, despite the higher WUE in the stressed plants under water limitation, there was a reduction in photosynthetic rates caused by both stomatal (initial) and non-stomatal limitations, the latter indicated by the maintenance of *C*_i_/*C*_a_ and the reduction in carboxylation efficiency (Figure 2D,E). These changes may be due to different factors related to the limitation of the enzymatic activity of RuBisCo, the enzymes of the Calvin cycle [34], or even the depletion of ATP and NADPH in the photochemical phase [35]. Similar responses have been observed in beans with *C*_i_ accumulation in leaf mesophyll and a reduction in CO_2_ assimilation, indicating non-stomatal limitations [36], and in tomato plants with a reduction in the activity of enzymes involved in the CO_2_ fixation process [37], due to low *A*/*C*_i_, grown under low-water conditions.

The increase in the ETR/*A* ratio (Figure 3F) indicates electron dissipation by alternative sinks, such as cyclic electron flow [38] or the water-water cycle [39], as the quantum efficiency of PSII was maintained (Figure 3C). However, less ATP and reduced energy were produced and utilized in the Calvin cycle for CO_2_ fixation.

Despite the decrease in photosynthetic activity, we observed no photoinhibition, as evidenced by the maintenance of the *F*_v_/*F*_m_ ratio and the lack of a significant increase in thermal dissipation or minimum fluorescence throughout the stress period (Figure 3A,B). Thermal dissipation is directly related to the xanthophyll cycle, in which activation of violaxanthin *de*-epoxidase during the conversion of violaxanthin to zeaxanthin serves to prevent overexcitation of PSII and protect against damage from excessive light [40,41]. 

In addition, the demand for photoassimilates is higher during the reproductive stage due to the high demands of flowering and fruiting [42]. We observed the maintenance of total soluble sugar and reducing soluble sugar content in the leaves of *R. sativus*, even under water deficit conditions (Figure 4A,B). The maintenance or increase in sugar content under drought conditions is due to the degradation of starch [43], which provides energy and carbon to plants [14]. The carbon derived from carbohydrate degradation in the GF stage was probably utilized for grain production [19,29], as bark dry matter and total pod dry matter were maintained at similar levels in drought-treated *R. sativus* plants as in control plants (Figure 5A,B). The bark prevents premature seed death by protecting the seeds from stress and serving as a source of assimilation [29].

The translocation of photoassimilates into the pods and the remobilization of the pods for grain development are important mechanisms for adaptation to water deficit [44]. Nevertheless, the decrease in photoassimilate production in leaves and the change in carbon distribution in the plant during the GF stage impaired the fractionation of carbohydrates between organs, leading to a decrease in 100-grain matter and productivity of *R. sativus* under water deficit conditions (Figure 5C,D). Damage to grain size, weight, and yield was also observed in soybean plants in response to water deficit during the GF phase [13]. These results suggest that the sensitivity of *R. sativus* plants to water limitation is more pronounced during reproductive development, a critical phase that has also been described as hypersensitive in other crops [19,45]. 

The oil content of *R. sativus* grains decreased with water deficit, but their chemical composition was maintained (Figure 5E–H). Water deficits have been reported to lead to changes in the oil content and chemical properties of the oil [18,46], thereby affecting biodiesel quality [47]. The acidity, peroxide, and iodine content of *R. sativus* oil were maintained even under restricted water conditions (Figure 5F–H). The PV and IV values (Figure 5G,H) were similar to those observed by Ávila and Sodré [6], Chammoun et al. [7], and Oliveira et al. [1] in Brassicaceae species. They comply with the standards of the National Agency of Petroleum, Natural Gas and Biofuels (ANP) [48]. According to the ANP, the maximum values for acid, peroxide, and iodine in crude oil for biodiesel production are 0.50 mg KOH g^−1^, 10 mEq kg^−1^, and 120 g I_2_ 100 g^−1^, respectively. In this study, AV (Figure 5F), PV (Figure 5G), and IV (Figure 5H) remained at about 7 mg KOH g^−1^, 4 mEq kg^−1^, and 106 g I_2_ 100 g^−1^, respectively, even in plants subjected to water restriction. AV values above the recommended value for biodiesel use can be attributed to large amounts of polyunsaturated fatty acids in the oil of *R. sativus* seeds [6,10]. The chemical instability and susceptibility of vegetable oils with unsaturated fatty acids to hydrolysis leads to the release of free fatty acids [1,10].

Vegetable oils with free fatty acids, high degrees of unsaturation, and high viscosity are undesirable for fuel production, as these properties are transferred to biodiesel [9,47]. The quality of oil from *R. sativus* plants under water restriction was not affected by this stress, highlighting the potential of this species for biodiesel production, even when grown in regions with low rainfall, compared to other oilseeds such as sunflower [49,50], canola [51,52] and crambe [53,54].

## 4. Materials and Methods

### 4.1. Plant Material and Experimental Conditions

We conducted the experiment at the experimental station of the Goiano Federal Institute of Science and Technology—Rio Verde campus, Goiás, Brazil (17°48′28″ S, 50°53′57″ W) between July and October. The regional climate is classified as tropical savanna (Aw; Köppen 1931) with two clearly defined seasons: rainy summer (November to April) and dry winter (May to October). The average annual temperature and precipitation are between 20 °C and 35 °C and 1200 and 1800 mm, respectively. 

The soil was classified as a dystroferric red latosol (LVdf) with medium texture [55]. The chemical and water-physical properties of the soil at 0.00–0.20 m and 0.20–0.40 m are described in Table 1. Liming and fertilization of the soil were based on chemical analysis and species requirements [56].

To establish an adequate plant population, 20 seeds of *Raphanus sativus* L. var. *oleiferus* Metzg, cultivar CATI AL 1000, were sown per running meter of furrow at a depth of 0.02 m and used to establish the crop. After crop establishment (28 days after planting—DAP), we suspended the irrigation until the soil reached the critical tension of 20 kPa. Then, we initiated the treatments: water replacement at 100% (control), 66%, and 33% of soil moisture at field capacity (FC). We applied the irrigation using a dripline model with a nominal flow rate of 1.0 L h^−1^ and a drip spacing of 0.5 m. Irrigation demand was based on the predetermined critical tension and monitored using digital puncture tensiometry. We installed the tensiometers at depths of 0.10 m, 0.20 m, and 0.30 m parallel to the planting row, and we measured daily the soil matrix (Ψ_m_). The physical and hydraulic properties of the soil were determined using the Van Genuchten [57] soil water retention curve. We carried out the crop management measures as needed during the experimental period. We monitored the temperature and relative humidity using an automated meteorological station (Table 2) in the city of Rio Verde (Rio Verde University), and we obtained precipitation data from pluviometers installed in the experimental area (Table 2).

We experimented with a randomized block design with four replicates in a temporal split-plot scheme. The treatments consisted of three levels of water replacement: 100% (control), 66%, and 33% of FC in the subplots during the three phenological development stages: vegetative (VE), flowering (FL), and grain filling (GF). The experimental plots with an area of 8.0 m^2^ (2.0 × 4.0 m) consisted of four seed rows, each 0.50 m apart, and only the two middle rows were considered for measurement to avoid edge effects. About 15 days after sowing, we introduced a water restriction, which was maintained until the grain-filling phase and then suspended.

Analyzes of water relations, gas exchange, chlorophyll *a* fluorescence, chloroplast pigment, sugar, and proline (Pro) content were performed during the developmental stages vegetative (VE; 36 DAP), flowering (FL; 50 DAP) and grain filling (GF; 57 DAP). We carried out the physiological and biochemical assessments on three randomly selected plants within the cultivation area of each experimental unit. We evaluated the oil production components and chemical properties at the end of the crop cycle (120 DAP).

### 4.2. Water Relations

Pre-dawn leaf water potential (Ψ_w_) was measured using a Scholander pressure chamber (3005-1412, Soilmoisture Equipment Corp., Goleta, CA, USA). We determined the osmotic potential (Ψ_s_) of the leaves with a vapor pressure osmometer (5600, Vapro, Wescor, Logan, UT, USA), according to Pask et al. [58]. We determined The Ψ_s_ values using Van’t Hoff equation: Ψ_s_ = −R × T × C*_S_*, where R is the universal gas constant (0.08205 L atm mol^−1^ K^−1^), T is the temperature in degrees Kelvin (T °K = T °C + 273) and C*_S_* is the solute concentration (M) expressed in MPa (0.987 ≈ 1 atm = 0.1 Mpa). We determined the relative water content (RWC) by weighing the fresh matter (FM), the turgid matter ™, and the dry matter (DM) of the leaf discs and calculated as RWC (%) = (FM − DM)/(TM − DM) × 100, according to Barrs and Weatherley [59]. We measured the Ψ_w_ and RWC between 0400 and 0600 h and the leaves for the Ψ_s_ measurements between 0900 and 1000 h.

### 4.3. Gas Exchange

Gas exchange was measured in fully developed leaves in the middle third of the plant to determine the photosynthetic net assimilation rate (*A*, μmol CO_2_ m^−2^ s^−1^), stomatal conductance (*g*_s_, mol H_2_O m^−2^ s^−1^), transpiration rate (*E*, mmol H_2_O m^−2^ s^−1^) and the ratio between internal and external CO_2_ concentration (*C*_i_/*C*_a_). We calculated the water use efficiency as WUE = *A*/*E* and the instantaneous carboxylation efficiency as *A*/*C*_i_ (µmol m^−2^ s^−1^) [60]. We performed measurements between 0800 and 1100 h using an infrared gas analyzer (IRGA, LI-6400XTR, Licor^®^, Lincoln, NE, USA) under constant photosynthetically active radiation (1000 µmol photons m^−2^ s^−1^) and atmospheric CO_2_ concentration (*C_a_*) (~430 µmol mol^−1^), temperature (~25 °C) and relative humidity (48–65%).

### 4.4. Chlorophyll Fluorescence

We measured chlorophyll fluorescence parameters on the same leaf used for gas exchange measurements using a pulse amplitude modulation fluorometer (MINI-PAM, Walz, Effeltrich, Germany). First, we recorded the minimum (*F*_0_) and maximum (*F*_m_) fluorescence in dark-adapted leaves and calculated the potential quantum yield of PSII (*F*_v_*/F*_m_ = [*F*_m_ − *F*_0_]/*F*_m_). After prior light exposure, we applied a saturating pulse to determine the maximum fluorescence (*F*_m_′), steady-state fluorescence (*F*_s_), and initial fluorescence in light-adapted leaves (*F*_0_′ = *F*_0_/[*F*_m_ − *F*_0_/*F*_m_] + [*F*_0_/*F*_m_′]) and the effective quantum yield of PSII (Y_II_ = [*F*_m_′ − *F*]/*F*_m_′ [61]). We also used Δ*F/F*_m_*′* to estimate the apparent electron transport rate (ETR = Δ*F/F*_m_′.PAR.0.84.0.5) [62], where PAR is the photon flux (µmol m^−2^ s^−1^) on the leaves, 0.84 is the absorbed fraction of light incident on the leaves [63], and 0.5 is the fraction of excitation energy directed to PSII [64]. From these data, we calculated the non-photochemical quenching coefficient (NPQ = [*F*_m_ − *F*_m_′]/*F*_m_′) and the estimate of the ratio between ETR and photosynthetic assimilation rate (ETR/*A*) [65]. We carried out the measurements between 0700 h and 1100 h.

### 4.5. Electrolyte Leakage Rate

We measured cell membrane stability by the electrolyte leakage rate (ELR) in the leaf discs. We immersed the samples in ultrapure water and measured the initial conductivity (IC, µS/cm) after 24 h using a conductivity meter (CD-850, Instruthern, São Paulo, Brazil). Subsequently, we dried the samples in an oven at 100 °C for one hour to obtain the total conductivity (TC, µS/cm). We calculated the ELR as ELR (%) = [(IC/TC) × 100], according to Pimentel et al. [66]. We collected leaf discs at 0600 h.

### 4.6. Chloroplast Pigments

Pigment concentrations were determined by leaf extraction with dimethyl sulfoxide (DMSO) saturated with calcium carbonate, according to Ronen and Galun [67]. The leaf discs were incubated in the DMSO solution for 24 h at 65 °C in a water bath. The sample solution was measured at 480, 649, and 665 nm using a UV-VIS spectrophotometer (Evolution 60S, Thermo Fisher Scientific Inc., Needham, MA, USA). The concentration of chlorophyll *a* (Chl*a* = 12.19*A*_665_ − 3.45*A*_649_), *b* (Chl*b* = 21.99*A*_649_ − 5.32*A*_665_), and the total carotenoid content (Car = (1000*A*_480_ − 2.14C_a_ − 70.16C_b_)/220) were calculated according to Wellburn [68] and expressed by area. We collected leaf discs between 0400 h and 0600 h.

### 4.7. Proline

We analyzed the concentration of free proline according to Bates et al. [69], with modifications. We homogenized fresh leaf material in 80% ethanol to obtain the crude extract. We incubated the reaction solution, consisting of the crude extract, ninhydric acid solution (ninhydrin, glacial acetic acid, and 6 M orthophosphoric acid, 1.25:30:20 m/v/v), glacial acetic acid, and 125 mM glycine, at 90 °C. After 35 min, we stopped the reaction in an ice bath and added toluene to separate the proline. We measured the supernatants at 515 nm in a UV-VIS spectrophotometer and compared the absorbance values with the proline standard curve (0 to 100 μg mL^−1^). The results were expressed in μmol mg^−1^ fresh matter (FM).

### 4.8. Sugar and Starch

Fresh leaf material was immersed in 80% ethanol, incubated at 65 °C for 30 min, and then homogenized. We used the final extracts obtained from the supernatants after three extraction washes of the leaf material to determine the content of total soluble sugars, reducing sugars, and non-reducing sugars. The pellet was dried in an oven at 65 °C for 72 h to determine the starch content. 

The total soluble sugar (TSS) content was determined using the phenol-sulfur method of DuBois et al. [70]. The reaction solution containing an ethanolic extract, 5% phenol, and sulfuric acid (H_2_SO_4_) was kept in a water bath at 30 °C for 20 min. After reaching room temperature, we read the solution at 490 nm in a UV-VIS spectrophotometer, and the TSS was calculated using a standard sucrose curve (0–40 μg) and expressed as mg g^−1^ FM.

The reducing sugars (RS) were determined using the dinitrosalicylic acid method of Miller [71]. The reaction solution containing potassium sodium tartrate, sodium carbonate (Na_2_CO_3_), sodium bicarbonate (NaHCO_3_), sodium sulfate (Na_2_SO_4_), copper sulfate (CuSO_4_), ammonium molybdate (NH_4_)_2_MoO_4_), sodium arsenate (Na_2_HAsO_4_) and H_2_SO_4_ was read at 540 nm in a UV-VIS spectrophotometer. RS content was calculated using a standard glucose curve (0–50 μg) and expressed as fresh matter (mg g^−1^ FM). We calculated the non-reducing sugar (NRS) content using the difference between the total and reduced sugar content.

We determined the starch content (Sta) according to McCready et al. [72]. The dried pellets, obtained after sugar extraction, were resuspended in 52% perchloric acid, held for 30 min, and then measured at 490 nm. The starch content was calculated using a sucrose standard curve (0–50 μg) and expressed in mg g^−1^ FM. The total non-structural carbohydrate (TNC) content was calculated as TNC = TSS + Sta, according to Silva et al. [73].

### 4.9. Productivity Components

We evaluated the total pod matter (TPM), bark matter (BM), and grain matter (GM) of 100 pods. Plants were manually harvested in a 1-m^2^ area of each plot and air-dried in an oven to determine grain yield (GY). We extrapolated the GY to kg ha^−1^.

### 4.10. Chemical Characterization of R. sativus Crude Oil

The oil of *R. sativus* was extracted from the dried and ground grains (~100 g) with hexane PA in a Soxhlet extractor after heating for 8 h, according to Instituto Adolfo Lutz [74]. We separated the oil extracted from the solvent under reduced pressure in a rotary evaporator (TE-210, Tecnal, Piracicaba, Brazil). We calculated the oil content from the oil matter (OM) and the milled grain matter (GM) as OC (%) = [(OM/GM) × 100]. 

We analyzed the crude oil chemical properties volumetrically, including acid value (AV), peroxide value (PV), and iodine value (IV) according to AOCS [70] and IAL [75]. We performed triplicate measurements and read a blank value for each analysis. 

We determined the AV (mg KOH g^−1^ oil) from 1.5 g oil with potassium hydroxide (KOH) titration solution. We standardized the KOH titration solution with potassium dichromate in an acidic medium. We determined the PV (mEq kg^−1^ sample) from 1.0 g of oil with a titration solution of sodium thiosulphate (Na_2_S_2_O_3_). We determined he IV (g I_2_ 100 g^−1^ oil) in 0.1 g oil using the Hanus method with Na_2_S_2_O_3_ as tritant. For both PV and IV analysis, we previously tritant solution standardized with potassium dichromate (K_2_Cr_2_O_7_) in an acidic medium.

### 4.11. Statistical Analysis

We evaluated the water relations, physiological, and biochemical traits in the three developmental stages and water replacements. We used a two-way ANOVA to compare these factors (stage and water). The component production and crude oil characterization were evaluated only at the end of the experiment, and a one-way ANOVA was used to compare differences between water changes. To evaluate whether there is a separation of parameters considering the development stage and water levels for the water relations, physiological, and biochemical traits, we used principal component analysis (PCA). We used the same analysis to assess whether there was a segregation between the production and characterization of the crude oil and the water replacement treatments. The PCA was followed by permutative multivariate analysis of variance (PERMANOVA) using Euclidean distance to test for the significance of ordination using the “adonis2” command. We considered all data significant if *p*-values were <0.05, corresponding to 95% of the confidence interval. For ANOVA, PCA, and PERMANOVA, we used the “vegan” package [76] in the Rstudio program [77]. 

## 5. Conclusions

*R. sativus* plants showed drought tolerance mainly during their vegetative stage due to an adaptation of photosynthetic metabolism that allowed high stability of PSII and the complex antenna and, consequently, photochemical processes. The increase in Pro content served to prevent membrane damage and maintain the water content of the tissue. The GF stage was the most sensitive to water stress. Despite a slight decrease in grain yield and oil content, the oil produced by *R. sativus* had adequate chemical properties, indicating its potential suitability for biodiesel production.

## Figures and Tables

**Table 1:** Chemical and water physical properties of the soil in the experimental area. Rio Verde, GO, Brazil.

**Chemical Properties**											
Depth	pH	O.M.	P	K	Ca	Mg	Al	H+Al	S	CEC	V
(m)	H_2_O	(g kg^−1^)	(mg dm^−3^)	(mmol dm^−3^)					(mg dm^−3^)	(cmolc dm^−3^)	(%)
0.00–0.20	6.2	63.42	7.06	2.04	20.4	16.8	0.0	57.75	41.80	99.55	41.99
0.20–0.40	6.6	44.47	2.65	4.09	14.4	13.2	0.0	44.55	31.69	76.24	41.57
**Physical-aqueous properties**											
Depth	Granulometry (g kg^−1^)			θ_FC_	θ_PWP_	Sd	TP		Texture classification		
(m)	Sand	Silt	Clay	(%)	(%)	(g cm^−3^)	(cm^−3^ cm^−3^)				
0.00–0.20	458.3	150.2	391.5	51.83	30.50	1.27	0.55		Franco Argiloso		
0.20–0.40	374.9	158.3	466.8	55.00	31.33	1.28	0.51		Clay		

θ_FC_, field capacity (10 kPa); θ_PWP_, permanent wilting point (1.500 kPa); Sd, soil density; TP, total porosity; pH values in distilled water, P and K, Mehlich^−1^ extractor; O.M., organic matter; CEC, cation exchange capacity; V, base saturation (SB/CEC ratio).

**Table 2:** Monthly average values of climatic variables recorded during the experimental period. Rio Verde, GO, Brazil.

Month	Temperature			RH	Precipitation
	Maximum (°C)	Minimum (°C)	Average (°C)	(mm)	(mm)
July	28.4	14.2	20.5	57.0	2.3
August	31.7	15.7	22.7	44.0	0.0
September	33.8	18.8	24.8	54.0	2.1
October	32.3	19.1	24.6	53.0	15.5

RH—Relative humidity.

## Data Availability

Data is contained within the article.

## Supplementary Materials

The following supporting information can be downloaded at: https://www.mdpi.com/article/10.3390/plants13121583/s1, Table S1: Analysis of variance for physiological parameters between water replacement and vegetative stage. *F* and *p*-values obtained in ANOVA two-way analysis; Table S2: Analysis of variance for productivity parameters between water replacement. *F* and *p*-values obtained in ANOVA one-way analysis; Table S3: Pre-dawn leaf water potential (Ψ_w_, MPa), leaf osmotic potential (Ψ_s_, MPa), relative water content (RWC, %), photosynthetic net assimilation rate (*A*, μmol CO_2_ m^−2^ s^−1^), stomatal conductance (*g*_s_, mol H_2_O m^−2^ s^−1^), transpiration rate (*E*, mmol H_2_O m^−2^ s^−1^), ratio between internal and external CO_2_ concentration (*C*_i_/*C*_a_), water use efficiency (WUE), instantaneous carboxylation efficiency (*A*/*C*_i_, µmol m^−2^ s^−1^), minimum fluorescence (*F*_0_), potential quantum yield of PSII (*F*_v_*/F*_m_), effective quantum yield of PSII (Y_II_ ′), apparent electron transport rate (ETR), ratio between ETR and photosynthetic assimilation rate (ETR/*A*), non-photochemical quenching coefficient (NPQ), electrolyte leakage rate (ELR, %), total chlorophyll (Chl*a*+*b*), total carotenoid content (Car), proline concentration (PRO, μmol mg^−1^ FM), total soluble sugar content (TSS, mg g^−1^ FM), reducing sugars (RS, mg g^−1^ FM), non-reducing sugar sugar content (NRS, mg g^−1^ FM), starch content (Sta, mg g^−1^ FM), total non-structural carbohydrate content (TNC, mg g^−1^ FM), grain yield (GY, kg ha^−1^), total pod matter (TPM), bark matter (BM) and grain matter (GM) of 100 pods, oil content (OC, %), oil acid value (AV, mg KOH g^−1^ oil), oil peroxide value (PV, mEq kg^−1^ sample) and oil iodine value (IV, g I_2_ 100 g^−1^ oil) in *Raphanus sativus* plants exposed to three water levels: 100% (control), 66% and 33% of field capacity and at three phenological stages of development: vegetative (VE), flowering (FL) and grain filling (GF).
