# Supplementary material for: Morpho-Physiological Traits and Oil Quality in Drought-Tolerant Raphanus sativus L. Used for Biofuel Production
Source: Plants (Basel). 2024 Jun 7;13(12):1583. doi: 10.3390/plants13121583 (PMC11207979; doi:10.3390/plants13121583)
Supplement: Supplementary file 1 [file plants-13-01583-s001.zip › plants-2999536-supplementary/Table S1.pdf]

Table S1. Analysis of variance for physiological parameters between water replacement and vegetative stage. *F* and *p*-values obtained in ANOVA two-way analysis.

Trait	Water replacement		Stages	
	F-value	*p*-value	*F*-value	*p*-value
Predawn leaf water potential (Ψ_w)	13.57	<0.0001	96.63	<0.0001
Leaf osmotic potential (Ψ_s)	1.24	0.3	4.47	0.02
Relative water content (RWC)	1.35	0.27	9.22	<0.001
Photosynthetic net assimilation rate (*A*)	0.74	0.49	33.56	<0.0001
Stomatal conductance (*gs*)	20.69	<0.0001	74.77	<0.0001
Transpiration rate (*E*)	22.31	<0.0001	59.08	<0.0001
Ratio between internal and external CO_2 concentration (C_i/C_a)	2.11	0.14	6.57	0.005
Water use efficiency (WUE)	9.28	0.008	6.46	0.005
Instantaneous carboxylation efficiency (A/C_i)	0.73	0.49	28.43	<0.0001
Apparent apparent electron transport rate (ETR)	3.07	0.06	12.18	0.0002
Ratio between ETR and photosynthetic assimilation rate (ETR/*A*)	0.94	0.4	26.65	<0.0001
Minimum fluorescence (F_0)	1.31	0.29	22.76	<0.0001
Potential quantum yield of PSII (Fv/Fm)	3.08	0.06	1.53	0.23
Effective quantum yield of PSII (Y_{II})	0.06	0.94	6.75	0.004
Non-photochemical quenching coefficient (NPQ)	4.91	0.01	0.73	0.49
Total chlorophyll content (Chl*a*+*b*)	0.17	0.84	3.02	0.06
Carotenoids (Car)	3.93	0.03	4.18	0.03
Electrolyte leakage rate (ELR)	5.11	0.01	18.99	<0.0001
Total soluble sugars (TSS)	1.75	0.19	10.48	0.005
Reducing sugars (RS)	0.29	0.75	11.76	0.0002
Non-reducing sugars (NRS)	1595	<0.0001	0.27	0.77
Starch (Sta)	28.18	<0.0001	66.96	<0.0001
Total non-structural carbohydrates (TNC)	18.62	<0.0001	56.13	<0.0001
Proline (Pro) content	15	<0.0001	191.37	<0.0001
