# Supplementary material for: Morpho-Physiological Traits and Oil Quality in Drought-Tolerant Raphanus sativus L. Used for Biofuel Production
Source: Plants (Basel). 2024 Jun 7;13(12):1583. doi: 10.3390/plants13121583 (PMC11207979; doi:10.3390/plants13121583)
Supplement: Supplementary file 1 [file plants-13-01583-s001.zip › plants-2999536-supplementary/Table S2.pdf]

Table S2. Analysis of variance for productivity parameters between water replacement. *F* and *p*-values obtained in ANOVA one-way analysis.

Productivity	Water replacement	
	F-value	*p*-value
Total pod matter (TPM)	1.31	0.32
Bark matter (BM)	0.67	0.54
Grain matter (GM)	24.33	0.0002
Grain yield (GY)	10.47	0.004
Grain oil content (OC)	4.42	0.02
Acid value (AV)	3.89	0.03
Peroxide value (PV)	1.17	0.32
Iodine value (IV)	3.14	0.06
